# Family-centered approach in Early Childhood Intervention of a vulnerable population from an Ecuadorian rural context

**DOI:** 10.3389/fpsyg.2023.1272293

**Published:** 2023-12-01

**Authors:** Marcela Frugone-Jaramillo, Marta Gràcia

**Affiliations:** ^1^Universidad Casa Grande, Guayaquil, Ecuador; ^2^Department of Cognition, Development and Psychology of Education, Universitat of Barcelona, Barcelona, Spain

**Keywords:** family-centered practices, family quality of life, sense of family competence, natural environments, rural families of children with disabilities

## Abstract

**Introduction:**

Studies about the implementation of the Family Centered Practices approach in Early Childhood Intervention refer as outcomes that have an impact on the Quality of Family Life, on children’s development, and also on family empowerment. In Ecuador, despite an absence of Early Childhood Intervention policies and programs, a university has developed training in Family Centered Practices for graduate students. A formative component is to implement a Routines Based Model with families of children with disabilities. The aim of the study is to analyze the impact on the Family Quality of Life, children’s development and self-perceived competence of families after the Routines Based Model has been implemented in their natural environment.

**Method:**

Eight families from a rural area and their children with disabilities were included in the study. The Family Quality of Life Scale-Early Childhood Intervention and the Screening of the Battelle Developmental Inventory were applied at the beginning and end of the process. A qualitative interview established the family perspective upon the outcomes in their family and their children.

**Results:**

There is evidence of a significant increase in the families’ Quality of Life and in the children’s development at the end of the process. At the interview the families declared themselves more competent to understand and contribute to the development of their children.

**Discussion:**

The results provide knowledge of the implementation of a Routine Based Model in vulnerable contexts. Also contributes in the understanding of the family perspective on the outcomes and perceived benefits for the children and the family itself as a measure of quality of the intervention and training. Implications of the results for initial and ongoing training of early care professionals with vulnerable populations are discussed.

## 1 Introduction

Studies on implementing the Family-Centered Practices (FCP) approach in Early Childhood Intervention (ECI) identify the key outcomes as impacting on family quality of life (García-Grau et al., 2019), child development (McWilliam, 2016) and family empowerment (Fernández et al., 2017). In Ecuador, a university has developed training in PCF for postgraduate students, despite the lack of ECI policies and programs. As part of the training, a routine-based model (RBM) (McWilliam, 2016) has been implemented with families of children with disabilities. The aim of the study is to analyze parents’ perceptions of the impact of the intervention on family quality of life, child development and families’ self-perceived competence following the RBM delivered in a natural environment.

### 1.1 Family centered practices

Early Childhood Intervention (ECI) is a relatively young discipline (García-Sánchez et al., 2014). In the last decade, it has undergone a process of transformation with regard to the aims, practices and objects of intervention (Dunst, 2000; Gràcia et al., 2020). A radical change has been proposed, such as placing the family at the center of intervention (Espe-Sherwindt, 2008; Giné et al., 2009; Guralnick, 2017). Instead of an approach focused exclusively on the child and his/her deficit, with interventions poorly articulated between different professionals and with little family participation (García-Sánchez et al., 2014; Frugone-Jaramillo et al., 2020). This means responding to the needs identified by the family (Bamm and Rosenbaum, 2008; Briar-Lawson et al., 2012), in a treatment based on dignity and respect (Espe-Sherwindt, 2008). To achieve this, it is essential to consider the contextual circumstances, concerns and resources that families have or need beyond caring for their disabled children (Allen and Christopher, 1996). This new paradigm suggests that the most effective interventions are those that take place in the natural environment of the child and their family to achieve wellbeing for the whole family (Swanson et al., 2011; Espe-Sherwindt and Serrano, 2016; Dunst et al., 2017). Providing families with the knowledge and skills they need to create a stimulating environment for their child and for the whole family (Espe-Sherwindt, 2008; Dunst et al., 2017) is an important component of this.

### 1.2 Family-centered practice outcomes for families

This paradigm shift involves developing interventions that focus on outcomes that benefit the whole family, rather than just child development or family satisfaction, which have been used as measures of the quality of outcomes of ECI programs. The need to define what are family-friendly outcomes of a family-centered intervention is highlighted by Bailey et al. (2006). For these Frugone-Jaramillo et al. (2020), family satisfaction is primarily an indicator of family satisfaction with the therapy and not necessarily with the outcomes, which is what should really define intervention effectiveness. A more open-minded view of what families can report as benefits makes it possible to work towards a substantial improvement in the relationship between professionals and families. This will be reflected in a wider range of outcomes, which can include all the members of this environment: older or younger siblings, grandparents, aunts and uncles, etc. (Bruder and Dunst, 2005; Mas et al., 2016). Therefore, when considering children’s developmental outcomes, it is necessary to broaden the vision to include family outcomes based on the competencies they might develop (Dunst and Bruder, 2014; Gràcia et al., 2020; Escorcia-Mora et al., 2022; Frugone-Jaramillo and Gràcia, 2022).

### 1.3 Family quality of life as outcomes from family-centered practice

Improving family quality of life (QoF) (Giné et al., 2009; Gràcia et al., 2020; Verger et al., 2021) is defined as one of the central objectives of family-centered interventions. Family Quality of Life is a construct that recognizes the family’s ability to self-perceive different subjective and objective aspects of their quality of life. The concept of family quality of life also refers to a shared sense of family well-being and is therefore composed of subjective indicators (Balcells-Balcells et al., 2011). The improvement of family quality of life (QoF) (Giné et al., 2010; García-Grau et al., 2018; Verger et al., 2021) is defined as one of the central objectives of family-centered interventions. Family quality of life is a construct that recognizes the family’s capacity for self-perception of different subjective and objective aspects of their quality of life. It is therefore composed of subjective and objective indicators that are perceived individually and collectively by different family members (Giné et al., 2010). For Brown et al. (2009), families perceive the quality of family life when: (a) they identify goals and work to achieve them, (b) they are satisfied with their achievements, (c) they have aspirations for their lives, (d) they are satisfied with their achievements, and (e) they have aspirations for their lives and strive to achieve them.

The Early Intervention Family Quality of Life Scale (FQoLS-ECI), developed by McWilliam et al. (2009) and translated into Spanish by García-Grau and McWilliam (2014), is used in ECI. Research using this tool in different countries shows that it is valid for family self-assessment of FQoL at baseline and outcome (García-Grau and McWilliam, 2014; García-Grau et al., 2018; Escorcia-Mora et al., 2022; Frugone-Jaramillo and Gràcia, 2022).

### 1.4 Family empowerment

Family empowerment is another goal to be achieved through a family-focused intervention (Allen and Christopher, 1996; Dempsey and Keen, 2008; Fernández et al., 2017). This requires a change in the way family members are perceived by professionals. A positive view must prevail, in which they are seen as equals with positive attributes, capacities and resources (Allen and Christopher, 1996; Pereira and Serrano, 2014). Another concept of family empowerment refers to identifying, promoting or developing family and child capacities, creating positive perceptions of family control over daily family life (Dempsey and Dunst, 2004; Trivette et al., 2010). Family competence develops when families are able to identify their strengths and improve their decision-making skills, optimizing their sense of competence (McWilliam et al., 2009; Escorcia-Mora et al., 2022).

Based on the development of these competences, the literature points to the importance of including family empowerment as a goal of ECI, which does not happen when the intervention is directed exclusively at the child (Trivette et al., 2010; Fernández et al., 2017). The empowerment of the family is related to the type of collaborative practice that professionals develop with the family. Adopting participatory practices has been documented to increase the effectiveness of interventions with children and families (Dempsey and Dunst, 2004). However, the literature also suggests that the adoption of participatory practices is complex for practitioners (Mas et al., 2016; Maia et al., 2017). Translating the philosophical principles of PCF into practice enables us to understand, on one side, the richness of the proposal and, on the other, the complexity of its operationalization, since it implies both a change in the structure of services and sensitive and qualified professional training to implement it (Bamm and Rosenbaum, 2008; Woods and Lindeman, 2008; Frugone-Jaramillo et al., 2020; Oude-Matmann et al., 2022).

### 1.5 Family-professional collaboration and the routine based model

Working with families requires professionals collaborating in ECI teams within a transdisciplinary framework (García-Sánchez et al., 2014; McWilliam, 2016). Practitioners need to be empathetic and sensitive to family diversity, understood as diversity of social construction, social class, history, social and economic conditions and origin (Pretis, 2006; Swafford et al., 2015). Professional competencies related to cultural sensitivity and respect for cultural and socio-economic differences are particularly necessary to be developed when working on PCF-based interventions in rural areas. This is even more the case when there is still a lack of literature on the skills, support and resources needed to offer PCF to families with diverse structures, living in conditions of extreme poverty and in rural areas (Swafford et al., 2015).

The RBM developed by McWilliam (2010, 2016) takes the principles of FCP and makes them operational through a process of developing tools to carry out the essential steps of a family-focused intervention: (a) identification of family needs through the ecomap and routine interviews, (b) individual family support planning with goals embedded in everyday life, (c) a single professional accompanying families, supported by a transdisciplinary team, (d) home visits based on coaching, (e) a series of checklists that help the professional team to evaluate their intervention.

### 1.6 Involvement and perspectives of families in a FCP intervention

At the same time, it was important to gather the perspectives of families of children with disabilities. Previous experiences of specialist care had been deficit-focused, disjointed and clinic-based, with few opportunities to adapt to the demands of everyday life, and with little parental involvement (Frugone-Jaramillo and Gràcia, 2022). The needs of the family have been left out of traditional interventions (Giné et al., 2003, 2009; Woods and Lindeman, 2008). While research has been developed on family perceptions of family-centered interventions (Hugh-Scholes and Gavidia-Paynes, 2016; Costa et al., 2017) and outcomes on family relationships and competence (Swafford et al., 2015; Hugh-Scholes et al., 2019; Escorcia-Mora et al., 2022), more research is needed on family perceptions of interventions and family outcomes from a qualitative approach (Bamm and Rosenbaum, 2008). Findings of these studies show that parents experience less stress and are better able to make decisions and take action for the benefit of their children and the family. Parents also highlighted as common features feeling listened to, having received treatment based on kindness, sensitivity and respect, and having a professional available to listen and support them. These results are consistent with research on the sense of family competence perceived by families of preterm infants (Escorcia-Mora et al., 2022), the collaborative relationships established in a family-centered intervention for families in a rural area of Manabí (Frugone-Jaramillo et al., 2020), the perception of how professional support promotes the development of competencies in parents of preterm infants oriented towards the acquisition of language and communication in preterm infants (Frugone-Jaramillo et al., 2020). This research was conducted in Ecuador, specifically at the Ecuadorian university that pioneered PCF training and implementation in Ecuador.

Regarding levels of parental involvement in FCP interventions, several studies conclude that families become proactive in interventions when more participatory practices are used (Frugone-Jaramillo et al., 2020; Vanderkerken et al., 2021; Mas et al., 2022). Family empowerment is a central dimension to be developed in an intervention that embraces the principles of FCP, understood as the ability of families to make informed decisions for their children and for themselves as a family. Empowerment should be the goal of professionals in their work with families. However, it is an issue that requires a lot of support in order for early childhood professionals to be able to implement empowering actions effectively (Rouse, 2012).

### 1.7 Families of children with disabilities in Ecuador and ECI systems

According to the ecological approach to development (Bronfenbrenner, 1987), environmental conditions such as poverty are stressors that affect parental capacity and child development. In the case of children with disabilities living in rural areas, environmental constraints increase vulnerability (Ullmann et al., 2020). For caregivers, access to specialist programs and services is difficult, resulting in fewer opportunities for appropriate development [World Health Organization & World Bank (WHO), 2011]. The worries of the family are increased by the conditions of increased poverty, difficulties in mobilization and reduced access to specialized services (Quezada and Huete, 2017). At the same time, poverty increases due to the demands of caring for the family member with a disability and because one family member is usually forced to lose an income-generating job [World Health Organization & United Nations Children’s Fund (UNICEF), 2013; Ullmann et al., 2020].

In Ecuador [National Institute of Statistics and Censuses (INEC), 2019], 40% of the rural population lives in extreme poverty. This means that a poor person in the rural sector has a sporadic income that depends on agricultural activities. He or she usually has little schooling or education and may belong to an indigenous village. Their meager income is spent on food, which is not grown, and on the small amounts of support required by the free social services, such as money needed to celebrate festivals or to clean up the community. Chronic child malnutrition is widespread. 28% of children are malnourished [National Institute of Statistics and Census (INEC), 2018].

In Latin America, including Ecuador [DeWaal Foundation (FdW), 2022], there is a lack of ECI systems. Thus, Latin America and Ecuador are indebted to families in need of early childhood services (Guralnick, 2015).

While RBM has been adopted by several countries, there is little literature about interventions developed in vulnerable contexts (Swafford et al., 2015). This research is a contribution to the understanding of the development of culturally sensitive intervention based on RBM, as suggested by Division for Early Childhood of the Council for Exceptional Children (DEC) (2020). Child and family outcomes are key aspects in assessing the effectiveness of an intervention, which is presented in this research. It includes quantitative perspectives in terms of developmental and family quality of life scales, and a qualitative perspective by giving voice to families. Bernheimer and Weisner (2007) argue for the coherence of family-centered research in which the voice of the family should be heard.

This article provides a perspective on family outcomes from a research into reflective practice-based training for Master’s level child development students in the routine-based model. The results of the intervention with eight families of children with disabilities living in a rural area on the coast of Ecuador are reported. The article explores the perceptions of the families with regard to: 1) the results of the RBM in the quality of life of the families, 2) the results of the RBM in the development of their children, 3) the perceptions of the families about their participation in the RBM.

## 2 Methods

### 2.1 Design

The interest in a deeper understanding of the processes involved in this training experience, which takes place in a specific situation and context (Neiman and Quaranta, 2006; Álvarez and Fabián, 2012; Urra et al., 2014), led to the development of a case study focusing on the training experience and its outcomes. The case study, as a qualitative method, allows the integration of quantitative data (Neiman and Quaranta, 2006) that complement the understanding of the case, with a quantitative perspective prevailing.

### 2.2 Context of the study

This research was conducted as part of the master’s thesis of four students in an Ecuadorian university’s master’s degree in early childhood development. The students were early childhood educators. They were trained and accompanied by two teachers who were guides of the intervention process. All four students live in the province of Manabí, about 4 hours away from Guayaquil. They applied for the project and were accepted because they practice educational inclusion in their teaching. They signed an informed consent form as an acceptance of their participation in the research on this experience.

### 2.3 Participants

Eight families of children with disabilities or at risk in a rural sector of the coastal zone of Ecuador participated in this study. Six of the families participating in this study were recruited through contact with a public special school. Every 2 weeks their children received therapy. Two of the families were in more remote rural areas and could not afford treatment.

The inclusion criteria were: a) families with children with disabilities and/or at risk between 0 and 6 years of age; it was not necessary for their children to have a diagnosis due to the difficulties in accessing specialized services in the country, b) families with a medium-low socio-economic level, and c) families interested in participating in a six-month intervention and willing to sign the informed consent form (Tables 1, 2).

**Table 1:** Sociodemographic information about the 8 families participating in the research.

Families Codes	Children		Families				
	Condition	Age	Families	Parents occupation		Parents Education	
			Composition	Mother	Father	Mother	Father
F1	Down’s syndrome	5 years, 3 months	Mother, father, three brothers	Housewife	Farmer	No Education	No Education
F2	Cerebral Palsy	4 years, 7 months	Mother, grandmother, grandfather	Housewife	Farmer	Secondary School Completed	Secondary School Completed
			5 aunts and nieces				
F3	Child on the autism spectrum	3 years	Mother, father, brother, aunt	School Teacher	School Teacher	University education	University education
F4	Microcefalia	4 years	Mother, father, 2 sisters	Housewife	Soldering iron	University education	Secondary School Completed
F5	Laron’s syndrome	3 years 1 month	Mother, father, sister, uncle	Housewife	Construction worker	Elementary school	Elementary school
F6	Physical DisabilityEpilepsy	4 ages 5 months	Mother, father, brother	Housewife	Farmer	None	Elementary school
F7	Hand and facial malformations	4 years 4 months	Mother, father, sister	Housewife	Construction worker	High School studies	Elementary school
F8	Child on the autism spectrum	2 years 5 months	Mother, grandmother, grandfather, aunt, one niece	Secretary	Merchant	University education	Elementary school

**Table 2:** Relationship between specific research questions and specific interview questions.

Specific research aims
1.Identification of families’ perceptions of the impact of the student intervention on family quality of life and feelings of parental competence 2. To compare the children’s development before and after having implemented the PCF 3. To analyze the families’ perception of the intervention and of the developmental progress of their children

Specific research questions	Specific interview questions
Objective 5	
To analyze the families’ perceptions of the intervention and of the developmental progress of their children.	¿What were your concerns about your child and family at the start of the intervention? What did the family expect (for the child and for themselves) when the intervention started? Discuss the child’s assessment results with the family. Compare the baseline and endline scales. (There is likely to be little development between the two)
Objective 4	
Comparison of children’s development before and after the implementation of the PCF by the Masters students	Ask them what they, as the primary caregiver, think has happened to their child in this process Reflect with the parents or carers in what area has their child improved? In what area of development is there a need for further work? What were the difficulties in the areas where there were no major achievements?
Objective 3	
To identify families’ perceptions of the impact of the student intervention on the quality of family life and feelings of parental competence	Discuss the goals achieved in order of priority. Have a look at the objectives that you have been working on What have you achieved for each goal (presented in turn)? How did you achieve it? What has been the involvement of the family in the achievement of the goal? How was the goal incorporated into the family routine? What do you still need to work on in relation to this goal?
Objective 5	
To analyze the perceptions of the families about the intervention and the developmental progress of their children	In addition to the goals achieved with the child, which other family member has benefited from this intervention? (What competencies as a family/parent have they acquired? Analyze the Family Quality of Life scale at the beginning and now, how do they feel now?)
Objectives 3 and 5	
To determine families’ perceptions of the impact of the student intervention on family quality of life and sense of parental competence To analyze how families perceived the intervention and their children’s developmental progress	During the intervention we have asked you to record the process with photographs. You will be asked to choose 4 or 5 photographs that give us an idea of what the process has been like and/or what your family and child have achieved in the process Explain each photo: what does it represent, why do you think it can represent the process we went through?
Objective 5	
To analyze the perceptions of the families about the intervention and the developmental progress of their children	What have you learned as a family from the intervention? What did you not like about the intervention?

### 2.4 Instruments

The Family Quality of Life Scale-AT (McWilliam et al., 2013) and the Battelle Developmental Inventory Screening (Newborg et al., 2011) were administered at the beginning and end of the intervention to have a pre- and post-intervention baseline. In addition, a semi-structured interview was conducted with each family at the final session of the intervention. The students were trained in the use of the three instruments.

#### 2.4.1 The family quality of life scale in ECI

This Scale was developed by McWilliam et al. (2009) and translated into Spanish by García-Sánchez et al. (2014). The scale has 39 questions that are answered on a Likert scale with values from one to five, with one being “Inadequate” and five being “Excellent.” Its items address four dimensions: Access to Information, Family Relationships, Family Functioning and General Satisfaction. It is analyzed quantitatively. It is designed to be completed by parents.

#### 2.4.2 The Battelle Developmental Inventory Screening

The Battelle Inventory is a developmental assessment battery for children aged 0–8 years (Newborg et al., 2011). It evaluates personal and social, adaptive, motor, cognitive and communicative domains. It was chosen because it can be administered by teachers according to the profile of the students. Screening Assessment was used. The advantage of the Battele Screening Test is that it saves time without compromising reliability, allowing assessment in all areas of development. The assessment requires the child to complete tasks requested by the assessor according to the child’s developmental area and age. Students were trained to apply it to the children they would be caring for.

#### 2.4.3 Semi-structured interview

This qualitative technique encourages individual dialog with each family to elicit their perceptions of the routine intervention process and the impact of the intervention on the development of the children and their families. It has been developed by the researchers and used in similar research. The end-of-process interview has been developed by researchers and used in other studies (Frugone-Jaramillo et al., 2020; Frugone-Jaramillo and Gràcia, 2022). The interview consists of four moments: a first moment explaining that the interview concludes the intervention process, the presentation of the results at the level of the family’s quality of life and the child’s assessment, which serves to reflect on the changes in the child and the family, and the commitment to maintain the objectives once the intervention is completed. The families were informed that this would be the last visit of the intervention. All the families answer the same questions. The students ask the families to take pictures about diverse circumstances than represents changes in their children and families given by the intervention. The photography elicit the families to speak about the intervention.

During the interview, the functional goals that have been worked on are discussed and the achievements are analyzed. A reflection is made on what has been done and how it is linked to the principles of the PCF. The results of the initial and final developmental assessment are presented. The family’s perceptions of other aspects of development not covered by the scale are collected. Similarly, the results of the Family Quality of Life Scale and the main aspects of self-perceived quality of life are discussed. Finally, they will be reminded about the skills they have developed to support their children and family in the future.

### 2.5 Data collection

#### 2.5.1 Students training

The four master degree students participating in this work were trained in the principles of PCF and the strategies of the routine-based model (McWilliam, 2010, 2016) through an accompaniment based on reflective supervision [Division for Early Childhood of the Council for Exceptional Children (DEC), 2020]. Each student implemented the RBM with two families under the supervision of two researchers of the Casa Grande University during 6 months.

#### 2.5.2 Implementation with families

The intervention with the 8 families lasted 6 months between the first contact with the families and the final interview. The intervention was developed in five stages. Initially, family members were contacted, invited to participate, signed an informed consent form, and administered a family QOL scale. Then, the stages suggested in RBM by McWilliam (2010, 2016) took place: a) Authentic assessment This involved the recognition of the formal and informal social support networks of the family, for which the ecomap was used, a chart that is designed by the family under the guidance of the student. The other authentic assessment tool is the Routine Based Interview (RBI), which involves a detailed tour of the family’s daily life. The student assisting the family to identify the aspects that they consider most important, which forms the basis for the development of the Individualized Family Plan (IFP) The next phase of the RBM intervention is the creation of the IFP, which builds on the priorities identified by the family and generates functional objectives, that means activities to be embedded in the daily life of the family and carried out by the family. The visit of a single student supported by the interdisciplinary team, has also been implemented. Two families have been assigned to each pupil. This is done on a weekly visit to review the progress made according to the Individualized Plan, as well as to discuss any concerns or progress that the family may wish to raise. The intervention ended with the administration of the Family Quality of Life Scale (McWilliam et al., 2013) and the Battelle Screening (Newborg et al., 2011). At the last visit, the final interview was carried out. The aim was to give the family the results of the intervention to reflect on them, and to communicate that the intervention has ended and reinforce the commitment of the family to continue working for their child and for the family quality of life.

The guiding researchers trained the students on the application of the RBM, the scales and the final interview. The interview was recorded with the permission of the families. All the intervention and data collection was carried out in the families’ homes.

### 2.6 Analysis

The FQoLS-ECI scores were compared at baseline and at the end of the intervention, and analyzed using descriptive statistics. The four factors that make up the scale were broken down into: a) access to information and services, b) family relationships, c) child functioning, and d) overall life satisfaction (García-Grau and McWilliam, 2014). The data collected through the Battelle Developmental Inventory Screening were also analyzed using descriptive statistics, on this occasion taking into account both the chronological age and the term age of the children, as well as the results of the baseline and term assessments. The analysis was supplemented with the non-parametric Wilcoxon signed rank test. The SPSS program was used.

The semi-structured interview with the families was transcribed verbatim by the professionals and reviewed by the researchers. To analyze perceptions of their children’s development and of QoLF, a deductive category system was created (Thomas, 2003), linked to the items of the two scales, considering that it was feasible that other subcategories could appear with the continuous reading of the data. Repeated reading of the interviews generated the subcategory “sense of family competence.” In terms of the families’ perceptions of the intervention, a subsystem of inductive categories was developed (Thomas, 2003), which was reviewed by the researchers individually and discrepancies and agreements in the categorization process were discussed to bring consistency to the subcategory system. When entering the data into Atlas Ti v. 22, some subcategories were removed or merged. Table 3 presents the categories and subcategories with which the final interview with the families was analyzed.

**Table 3:** Final family interview category system.

Categories	Definition	Subcategories
1. Perception of the impact of the intervention on their children’s development	Family narratives and perspectives about progress in different developmental domains of their children attributable to the intervention	1.1. Progress in social relations 1.2. Progress in autonomy 1.3. Progress in communication and language 1.4. Motor development progress 1.5. Understanding of disability 1.6. Perception of general improvement 1.7. Commitment to working on motor development 1.8. Commitment to work on inclusive education 1.9. Commitment to working in language
2. Impact of the intervention on the quality of family life.	Impact of the intervention in accordance with the four dimensions of family quality of life: access to information, family relationships, overall satisfaction. Child functioning is analyzed in the previous subcategory. The subcategory “sense of family competence” has been added	2.1. Access to information 2.2. Family relations 2.3. Overall Satisfaction 2.4. Sense of family competence
3. Understanding of the intervention and aspects of satisfaction with it	Recognize the core aspects that characterize RBM: family interaction, everyday environment, family empowerment	3.1. Activities embedded in daily routine 3.2 Ability to understand functional objectives 3.4. Achievement of functional objectives 3.5. Initial perception of the intervention 3.6. Relations with the practitioner

The results of the scales are complemented by the narratives that the interviews allow the participants to construct. This allows the data to be triangulated and the results to be deepened (Okuda and Gómez, 2005).

### 2.7 Ethical considerations

The participants, students and families, signed the informed consent and data protection was ensured. Ethical considerations were also taken into account and the family data were anonymized by replacing the names of the children and their relatives with a code for each family. This ensured confidentiality during the transcription phase C. The approval of the Research Ethics Committee of the Ecuadorian University was obtained.

## 3 Findings

### 3.1 Child development outcomes

Regarding the developmental level of the children as measured by the Battelle Inventory, in Table 4 it can be noted that at the beginning of the intervention all the children had a developmental age significantly below their chronological age. Also Table 4 shows that, in general, there was a significant increase in the developmental level of the 8 children at the end of the intervention.

**Table 4:** Developmental level as measured by the Battelle Inventory at baseline and at the end of the intervention.

ID	Chronological age at the start of the intervention	Initial assessment	Chronological age at the end of the intervention	Final assessment
Child 1	5.3	2.5	5.9	4
Child 2	4.7	0.6	5.3	0.10
Child 3	3.4	1.7	3.11	2.9
Child 4	4.11	0.8	5.5	1.1
Child 5	3.1	2	3.7	2.9
Child 6	4.5	2.2	4.11	3.2
Child 7	4.4	1.9	4.10	3.9
Child 8	2.5	1	2.11	1.8

The eight participating children showed significant developmental progress on the Battelle Developmental Inventory. The final evaluation shows that the children have made more than a year’s progress in less than 6 months of intervention. Only children 2 and 4 show a minimum progress equivalent to 6 months, i.e., the time of the intervention. This progress can be attributed to the intervention. These were children with very limited development, older than 4 years, and in their initial assessments they did not average 1 year of developmental age, i.e., their development was stagnant. Within 6 months of the intervention, they made progress.

The progress made in the different areas of the children’s development is detailed in Table 5. It is evident that all children showed significant gains at the end of the intervention in social, personal and adaptive functioning, and in the different dimensions of communication.

**Table 5:** Assessment with Battelle Development Inventory pre- and post-intervention by dimensions.

		Child 1	Child 2	Child 3	Child 4	Child 5	Child 6	Child 7	Child 8
Pers. social	Start	18	23	13	10	19	17	12	9
	Final	36	28	34	23	35	25	25	16
Adaptive behavior	Start	25	10	18	6	19	21	18	5
	Final	29	11	30	12	28	25	30	12
Gross motor	Start	12	2	10	4	6	5	7	4
	Final	15	3	16	6	16	10	17	7
Fine motor	Start	13	3	9	4	8	10	7	3
	Final	14	6	17	13	15	12	10	7
Motor	Start	25	5	19	8	14	15	14	7
	Final	29	9	33	19	31	22	27	14
Responsive communication	Start	8	3	9	6	9	9	8	2
	Final	13	7	14	8	11	10	13	4
Expressive communication	Start	4	3	4	5	5	7	7	2
	Final	7	4	12	4	11	7	13	2
Communication	Start	12	6	13	11	14	16	15	4
	Final	20	11	26	12	22	17	26	6
Cognitive	Start	20	9	9	7	21	22	17	4
	Final	27	10	25	12	25	12	25	8

### 3.2 Results of the quality of family life in early childhood intervention

The overall average on the Family Quality of Life scale in ECI before the intervention was 2.4, equivalent to *partially inadequate* out of a score of 5, equivalent to *excellent*. At the end of the intervention, there was an overall increase in the families’ perception of family quality of life. In all eight families the mean increased by 1.5 points, resulting in an overall average of 3.9, close to *very adequate*. Table 6 presents the average scores of the FQoL-ECI at the beginning and end of the intervention for the 8 participating families.

**Table 6:** Average scores on the Family Quality of Life Scale in ECI at start and end of intervention per family.

Families	Initial scores	Final scores	Difference
Familie 1	1.6	4.2	2.5
Familie 2	1.5	3.7	2.1
Familie 3	2.5	4.2	1.7
Familie 4	3	4.1	1.1
Familie 5	2.7	4.1	1.4
Familie 6	2.2	3.8	1.6
Familie 7	2.6	3.2	0.6
Familie 8	2.8	3.7	0.9

At the end of the intervention an increase of more than 2 points was observed in families 1 and 2. Family 1 went from an *inadequate* score of 1.6 to a *very adequate* score of 4.2. Family 2 started with a slightly lower score than F1, with an overall average of 1.5, and increased by 2.2 points to an overall average of 3.7, which is *adequate*. Families 7 and 8 have the smallest difference in score between the beginning and the end of the intervention. Family 7 has an increase of 0.6 and Family 8 an increase of 0.9. Both families move from *partly inadequate* to *adequate*. The other families had differences between baseline and endline scores of between 1 and 1.6 points, with average scores in the range of 3 to 3.9, which is close to *very adequate* as an assessment of their family’s quality of life.

Table 7 presents the results of the four dimensions of the Family Quality of Life Scale-ECI at the beginning and end of the intervention: access to information, overall satisfaction, child functioning and family relationships, both in overall values for each dimension and broken down by family as seen on Table 7.

**Table 7:** Dimensions of the Family Quality of Life Scale in Early Intervention Care, at the beginning and end of the intervention.

	Access to information		Child functioning		Family relationships		Overall satisfaction	
	Before	After	Before	After	Before	After	Before	After
Familie 1	1.5	4.5	1.4	3.2	2	4.4	1.5	4.5
Familie 2	1.3	3.8	1.2	3.2	1.9	3.9	1.5	3.8
Familie 3	2.6	4.4	1.7	3.7	3	4.3	2.6	4.4
Familie 4	3.5	4.5	1.7	2.9	3.3	4.3	3.5	4.5
Familie 5	2.1	4.1	3.9	4.1	2.6	4.1	2.1	4.1
Familie 6	1.9	3.9	2.5	3.5	2.4	3.9	1.9	3.9
Familie 7	2.6	3.3	2.3	3.1	2.8	3.2	2.6	3.3
Familie 8	3	3.8	2	3	3	4	3	3.8
Overall average	2.3	4	2.4	4	2.1	3.3	2.6	4

The quantitative analysis by dimensions of the FQoLS shows significant increases in the total of each of the four dimensions. In the dimension “access to information,” the score rises from 2.3 to 4, which is *very adequate*. The dimension “overall satisfaction” starts with 2.4 *inadequate*, and reaches 4, equivalent to *very adequate*. The child functioning dimension increases from 2.1 to 3.3, equivalent to *adequate*. The dimension of family relationships increases from 2.6 to 4, equivalent to *very adequate*. As can be seen in general, with the exception of the “child functioning” dimension, the other 3 dimensions have moved from an *inadequate* to a *very adequate* rating. The “child functioning” dimension is the one with the lowest average increase, starting at 2.1, and progressing to adequate with a score of 3.3, which implies going from *partially adequate* to *very adequate*. This implies a significant change in the family’s perception of their children’s capacities.

The Wilcoxon non-parametric test was used to analyze the average scores of the dimensions of the Family Quality of Life Scale in Early Intervention and the four dimensions of the same scale applied at the beginning and end of the intervention. The bilateral significance value obtained in all dimensions is 0.012. This value indicates that the increase in the final scores with respect to the initial ones is significant, which could be attributed to the intervention.

### 3.3 Qualitative findings

The results of the final family interview are presented below, covering aspects related to how families perceived their progress in Family Quality of Life. Families also analyze the children’s developmental progress as evidenced by the pre and post Battelle test scores. Finally, families’ perceptions of the intervention are presented.

#### 3.3.1 Perception of families about the FQoL

In the final interview, the families confirm the results that have just been presented with regard to the FQoL Scale. Table 8 presents the frequency with which families mention each subcategory related to Family Quality of Life.

**Table 8:** Results related to the analysis of dimension of FQoL in the interview.

Subcategories	Families								Total
	F1	F2	F3	F4	F5	F6	F7	F8	
Access to information	1	1	1	1	3	3	3	2	15
Family relationships	3	2	1	2	1	2	2	1	14
Overall satisfaction	2	2	1	2	2	2	6	6	22
Family sense of competence	2	2	1	2	1	6	4	4	22

Table 8 shows that all the families reports some expressions in the interview about the four aspects of the dimension of FQoLS. With regard to “access to information,” all of the 8 families make reference to it during the interview. For example, the mother of Family 1 just one time refers to progress in “access to information” in the following quote, that is very significant:

“The truth is that we have learned a lot about how to teach my child things, the truth is that I really wanted him to learn a lot of things, but sometimes I did not know how to teach him, I am very happy about what my son has learned and I hope that he will continue to learn more.”

In the following quote, the mother 1 reports an improvement in “family relationships” as the father and siblings become more involved with her son with Down syndrome and develop a “sense of parental competence.” This mother reports the following:

“In the house, I spend most of my time alone with my son, that is to say, he worked the most with me, but when the brothers or the father come in the afternoon, who sometimes comes early, they go down to pick up the clothes and tell him to do the activities, that is to say, they help their brother to get dressed. My other children helped, my father too, they sat at the table with him to help him when he wasn't feeling well or couldn't do it, the whole family helped.”

In this way, the different dimensions of the QoFLS are mentioned by the families. The phrase will usually have more than one category in it, because the families have recognized different elements that are complementary to their quality of life.

In the interview with Family 4, when they were asked about their perception of their quality of life, the mother emphasizes the improvement in “family relationships”: “*As a family we have become stronger*.” The mother of family 5, when asked how the intervention improved her family’s quality of life, replies about how important is for her the “access of information” about the development of their child:

“I have learnt a lot, I have really learnt a lot, I am not as desperate as I used to be, thinking that my son would not learn everything like the other children, but now I know that the fact that Juan is small does not mean that he does not have to learn other things. I can say that I know more about how children develop, grow and learn, that you don't always have to give them what they want, that they have to learn to wait. Look, you even gave me ideas and motivation to send my child to a regular school and that made me happy.”

Regarding the concept of a “sense of parental competence,” Family 2 says

*“Well*… *I think we are more united as a family now and we are able to work together for my son, that makes us happy, just to see my son learning things he didn't know.”*

“Overall satisfaction” with their quality of life is reflected in Table 9 for all families. Families 7 and 8 mention this sub-category most frequently in the interview. Family 6 expresses “overall satisfaction” and identifies “access to information” as a contribution to their family when asked how the intervention has supported their quality of family life. A greater “sense of family competence” and improved “family relationships” are acknowledged by the mother of Family 6. Despite poverty and other constraints, this long quote suggests a sense of “overall satisfaction.”

**Table 9:** Frequency of categorized quotes on families’ perceptions of their children’s developmental progress.

Subcategories	Families								Total
	F1	F2	F3	F4	F5	F6	F7	F8	
Progress in social relationships	1	2	2	3	2	6	4	1	21
Progress in autonomy	4	1	3	2	6	2	1	1	20
Progress in communication	2	0	2	2	2	1	0	2	11
Progress in motor development	0	3	0	8	5	5	0	2	23
Understanding of the disability	0	0	0	0	2	2	1	0	4
Perception of overall improvement	2	1	1	4	3	3	2	2	18

“As a family I have the feeling that we have learned, that we have more knowledge about the child's illness, that we are more aware that he will learn little by little and that we are the ones who should help him to learn more. Because here at home, this is the place where he lives and where he learns more and more every day. I also feel that we are more united as a family. For example, during this time we talk more with my husband about things, especially about the child's illness and about my other son, that they are different, but that we have to accept what God has given us. And no matter how hard reality can be sometimes, we have to be happy, and the truth is that we are calmer now with your help.”

The perception of family quality of life reflected in the scale is ratified in the quotes from the families obtained in the closing interview.

#### 3.3.2 Families’ perceptions of children’s development

Table 9 shows the frequency of categorized quotes on families’ perceptions of their children’s developmental progress.

Table 9 shows that in the interview all the families were aware of the changes in the development of their children. These are changes that are in line with the Battelle Development Inventory. When asked, the mothers specifically point out and describe the changes in their children’s development, which they also attribute to the characteristics of the intervention: professional guidance, strategies to achieve the functional goals and activities introduced into daily life.

The mother of Family 2 when was asked to describe her son’s progress and how it makes her feel, she replied:

“*My son has improved a lot in everything, he can already dress himself, he eats by himself, he plays with his brothers, the truth is I am happy because he is already doing things that he did not do before, so much so that now I do not worry about going to dress him, he does it by himself, he even changes his clothes more than twice a day, I have to talk to him to make sure that the clothes he has are still clean.”*

This quote shows progress in “autonomy,” “social interaction” and “perception of general improvement.” These are subcategories of the category “perception of the impact of the intervention on their child’s development.” The mother reports her child’s progress in the area of “communication and language” on the basis of “understanding the corresponding functional goal”:

“With this objective we have managed to know what my son wants, before we had to guess what he wanted, he would just point and shout and sometimes we didn't know what to do, but with the sign you taught us, every time Xavier wants something he goes to the sign and takes out the card of what he wants, for example if he wants water, he goes to the sign, takes out the card.”

Battelle’s results show minimal developmental progress in Child 2. This contrasts with his mother’s “general perception of progress in child development.” In the following quote she notes progress in “motor development”:

“As I told you at the beginning, my son has almost no toys, but with the ones we made for him we have managed to make him able to grasp, although for a short time and that is enough since my son did not grasp anything, that is, he could not hold anything in his little hands.”

Progress in the area of “social relations” is reflected in the following quote:

*“my little son already shares laughs and little games with my other son and with his cousins, although it was not easy, he was always kind of rough when someone other than me or my mummy (grandmother) approached him*.”

The progress in “motor development” is reflected in the following response in which she links the progress with the “achievement of the functional objective” set:

“With this objective if I am very happy, I think my son did more than I expected, now he can already roll over by himself, when he is on his back by himself he rolls over to his tummy, also he tries to sit more often.”

With regard to the progress in her son’s development, in the following quote the mother expresses a “perception of general improvement” in her son’s development, which she attributes to the intervention:

“At the beginning… he was a child who didn't speak and was afraid of being locked up and didn't even go to the toilet, but now he says some words: José, strawberry, daddy, mummy, we are on our way. He used to tell me that when I was going to drop him off, he’d say “let’s go” and that meant I was going to take him. He also says “bath,” “water,” I can even travel with him now, he doesn't cry anymore. We took him to the neurologist in Guayaquil and we had to get into a lift, we thought he was going to cry and he didn't. He was calm. He just claps his hands, that's what he does, and when he goes to the toilet he goes with me and sits down, before when I used to sit him down he would get constipated, now he sits down and does his business without any problems.”

In this narrative the mother gives specific examples of her child’s progress in “communicative language,” “autonomy” and “adaptive behavior.” Progress in “social relations” is expressed in the following quote *“Socially he has also improved because the kindergarten aunt (referring to the kindergarten teacher) tells me that he now joins his classmates and starts playing.”*

The Girl 4 is one of the children who had the most significant delay in their development. The results reflected in the Battelle Test are very low, but in the interview the mother reported a subcategory “perceived general improvement of the child,” which relates to the category “perceived development of her children due to the intervention”:

“You saw at the beginning that my baby was a restless child, very limp, she didn't take small steps, she lost her gaze, she didn't fit into our world. After you took over the case and your therapies helped my baby and the therapies she received at school, and I also gave her other therapies with stem cells, my child has improved a lot, as you have seen: she is already crawling, she is already in our world, she is already paying attention to what we say, what we talk about. My baby also takes small steps with me, she has gained muscle tone, she is very well, very active, she already talks, she says “mummy,” not always, but sometimes she says it. The truth is: Since you met her, there has been a great improvement!”

The previous quote was coded at the following subcategories: “progress in motor development,” “progress in communication and language” and “progress in social relations,” that are under the category “perception of their children’s development attributable to the intervention.”

#### 3.3.3 Family understandings of and satisfaction with the intervention

In the following episode, the mother reports on “progress in autonomy,” “progress in motor development,” “progress in communication and language” and the subcategory “progress in social relations,” also the subcategory “family participation.” This subcategories belonging to the category (see Table 10). “Understanding of the intervention and aspects of satisfaction with the intervention”:

**Table 10:** Frequency of citations of the subcategories on understanding of the intervention and aspects of satisfaction with the intervention.

Subcategoríes	Families								Total
	FI	F2	F3	F4	F5	F6	F7	F8	
Activities embedded in daily routines	5	6	2	1	3	2	3	7	33
Familiar Participation	3	5	6	5	2	4	4	3	32
Capacity to understand functional objectives	6	2	0	0	2	0	3	5	18
Achievement of functional goals	7	4	6	0	7	3	3	5	35
Traditional Practices	0	0	0	3	0	0	0	0	3
Initial perception of the intervention	1	1	1	1	1	1	2	1	9
Relationship with the professionals	0	3	3	4	0	4	3	5	22

“*Weeks ago when I was washing her mouth, she pulled out the brush and started washing and her father laughed so much, celebrating and recording, he told her to brush her tongue and she understood, took out her tongue and started brushing.*”

The mother of F5 relates changes in their child development that has been important for the mother, like improve in communication of his needs and waiting for attention, progress in these areas is reflected in the following quote:

*“My son has improved a lot in this objective. As I said before, if he asked for water or anything else and I didn't give it to him at that moment, mmmmmmm he would cry a lot and get desperate. Now he doesn't do that, sometimes he wants to do something*, *but I tell him, ‖You should wait a little bit‗, and calms down and he waits.”*

The quotation reflects progress in “communication and language” and “adaptive functioning.” It also reflects the mother’s communication and reassurance skills. This quote also reflects that what has been gained for the son has a relationship with the “functional goals” that have been worked on together during the intervention.

Table 10 shows these subcategories found in the different interviews. The subcategories relate to the families’ understanding of the different distinctive elements of a routine-based activity, as well as the quality of the relationships established by the professional and whether they recognize in the interview the type of practices developed by the professionals. 7 families mentioned “activities integrated into the routine” quite often when interviewed. F4 mentions it only once. In all the interviews, ‘family involvement’ in the child’s work is mentioned in similar numbers, between 2 and 5 times. 5 out of the 8 families have an “understanding of the functional goals” and have talked about them in the interviews. However, as a milestone in their children’s development, all the families interviewed talked about ‘achieving the functional goal’. When interviewed, Family 4 and Family 8 are the only ones who mention traditional practices in the intervention they refer to.

The mother of family 1, when asked about her initial perception of the intervention, described it as follows *“How do I tell you??… I believe that you are going to help me with my son, that you are going to give him therapies, just like when he went to the Foundation, and that I have to do therapies at home, I have to obey the students*.”

The mother of F2 describes her initial expectations of the intervention:

“At the beginning I thought it would be like those visits that the MIES (Ministry of Economic and Social Inclusion) people make, that they come to your home and look at the child, they do a few things and leave work for you to do, like manual work and other things, but when they started working with my son and my other son is also included and with my nephews, besides not only did you work with the child, you also made us (mother and grandmother) work, I began to realize that it was different from the work that the MIES people gave.”

The mother of F3 begins the interview by pointing out her initial perception of the intervention. She relates that she was notified at the special education school that she could have visits from students: *“Aunt Carolina from the special education school had already told me that a group of students from a university in Guayaquil were coming to visit me at my house to work with my son.”* The expectation of both the specialized education school and the family was that the work would be direct with the child. The mother reports that E2 explained the intervention in detail and their participation was noted.

The mother of the F4 recounts a scene similar to that of F3. In order to get involved in the intervention project, the teacher at the special school told her that the students could work with the girl. She was excited about the potential help and regretted the end of the intervention:

“The teacher, Caro, introduced us and said 'they are from a university in Guayaquil and they want to work with my little girl with therapies', I was happy because anything that helps my child and my family is welcome. I thanked God that they came to help my daughter, … that they can continue to help more children in this way, because you have been a great help to my family, especially to my baby.”

In this quote, the mother begins by feeling grateful for the possibility of direct therapy for her child. At the end of the intervention, she point out that the intervention has supported her daughter and also involved her family.

At the beginning of the intervention, the mother of F5 expected to receive therapies for her son: “*The expectation was that you would come to give therapies to help my kid improve his illness”*.

The mother of F6 explains her expectations of the intervention in relation to her previous experiences. These were focused on working on the deficit and on the child, with little involvement of the family:

“We and my husband thought that they were going to give our son therapies like they give him at school, that a person would do the therapies for the child and they would send us to do something here at home and that way they would help the child to learn things.”

The mother of the girl7 describes that she did not have positive expectations from the initial invitation to be part of the intervention:

“The truth and I am honest with you that at the beginning, when the teachers from the special school told us that a group of students wanted to do a project with children with disabilities, I thought it was a waste of time and I thought that since you are students you would give us homeworks on how to treat our daughter. Even with my husband we thought about not accepting the proposal, but after talking with my family in-laws they told us that we should accept the proposal to see how they could help us with our daughter.”

At first, the mother thought that she would receive a traditional intervention, focusing on the child’s deficit and giving instructions to the parents. This is how she describes her beliefs about the intervention:

“We also thought that you would just give therapies or sing or play like in some other programs, but after you explained to us what the Family-Centered Intervention Project is, where you supported our decisions and we worked hand in hand, as you say: a cooperative work.”

It is clear from the various quotes that families need support. However, they feel that many of the interventions they have received are not appropriate for working with them as a family and do not promote their children’s development. The families highlight the difference in the intervention they have received, which has included their interests and worked in collaboration with professionals.

## 4 Discussion

With regard to children development outcomes, these are linked to family empowerment, which is a central element driving change within intervention models incorporating FCP (Bailey et al., 2006; Hugh-Scholes and Gavidia-Paynes, 2016). The results of the developmental assessment that was applied to the children highlight the significant developmental gains that were achieved after 6 months of the intervention. In the case of the two children whose development was most compromised at the beginning of the intervention, they show progress corresponding to 6 months at the developmental age presented in the evaluation, which indicates that the intervention has generated progress in those children. In terms of their children’s development, all eight families report a “perception of general improvement” in their children that they believe that are in direct association with the intervention.

Literature emphasizes that the Quality of Life is considered one of the aims of ECI (García-Grau et al., 2019; Subiñas-Medina et al., 2022), and a quality factor of the ECI service (Subiñas-Medina et al., 2022). Over the course of the intervention, there were no significant changes in family income or caregiver employment. All families, regardless of parents’ level of schooling and socio-economic status, report improvements in their quality of life. These results contrast with the study by García-Grau et al. (2019) conducted with 250 Spanish families receiving ECI services for their children. In that study, the increase in QoLF was related to the disability status of the children or the level of schooling of the families.

Reviews of research in which family-based interventions have been implemented report benefits to families in terms of increased parental knowledge of general child development and improved understanding of their child’s condition (Hugh-Scholes and Gavidia-Paynes, 2016). Consistent with what is reported in the literature (Dunst et al., 2017; Fernández et al., 2017), these studies observe increases in psychological well-being, parental sense of self- competence, self-efficacy, sense of personal control and ability to make informed decisions. These aspects are found in the different dimensions of family quality of life perceived by the families in this study as a result of the intervention.

In the present study, there was an increase in all dimensions of family quality of life: access to information, family relationships, child functioning and overall life satisfaction. Responses on the Family Quality of Life Scale coincide with qualitative responses from the final interview. The concept of “sense of parental competence” was included as a subcategory linked to the Family Qualitiy of Life category. This was done in order to qualitatively analize the final family interviews. This change is supported by the literature review that links higher perceiver FQoL with higher perceived trust and parental competence (McWilliam, 2010; Fernández et al., 2017). This approach is in line with the findings of this study.

Arellano and Peralta (2015) in their qualitative study of parents’ perceptions of children with disabilities, highlight parents’ appreciation of access to information about their children’s condition, allowing them to intervene appropriately with their children. In this study ‖access to information‗ was highlighted for all the families as a relevant dimension of QoLF. It is mentioned in the interviews by all the families (Arellano and Peralta, 2015).

The family relationships dimension extends relationships to other family members such as parents, siblings, grandparents, cousins or other extended family members (Bailey et al., 2006). This includes working to increase the involvement of fathers in families. This is to ensure that the burden of caring for a child with a disability does not fall solely on the mother. In the case of this research, a greater involvement of family members was achieved for the benefit of the development of the children and the well-being of the mothers. That can be observed on the F2, they child have cerebral palsy, with whom the extended family members did not have adequate interaction. In an interaction based on play and on the recognition of the child’s abilities, the siblings and the cousins were able to interact with Child 2. Another relevant example is the improvement in sibling relationships. Being able to control the disabled sibling’s impulses and having greater parental presence and competence to supervise sibling play was reported by F6.

Concerning the category “Understanding of the intervention and aspects of satisfaction with it,” which is analyzed in the final interview with the families, the families recognized the importance of “activities integrated into the daily routine” in supporting their children’s development. This was illustrated by the 33 times this subcategory was mentioned in the interviews. They also recognized that the intervention increased “family participation.” This was reflected in 32 citations in the 8 interviews. Families could “understand functional objectives” and “work towards achieving functional objectives.”

The methodology, which involves qualitative data, as in the case of the final interview with the families, contrasting scales and means of evaluation with a more quantitative approach, makes it feasible to obtain the families’ perspective and a better understanding of the aspects that the families value and have gained from the intervention.

## 5 Conclusion

The results of this intervention strengthen the argument that FCPs provide and create opportunities that enhance, support and strengthen family functioning, i.e., impact on improved parental empowerment (Dunst and Trivette, 2009; Costa et al., 2017). Empirical evidence shows that family-centered interventions have a positive impact on children and their families (Dunst et al., 2013; Frugone-Jaramillo et al., 2020; Escorcia-Mora et al., 2022). Improving parents’ perceptions of their own abilities affects their ability to attend to and understand their children with disabilities’ behavior and promote activities that support children’s functioning across developmental domains (Hugh-Scholes and Gavidia-Paynes, 2016).

This research demonstrated that RBM is a viable approach when working with poor rural families. Based on the priorities identified by the families in the RBI, intervention plans were developed and incorporated into daily routines. There is evidence that families have been able to identify the features of the intervention, and that as a result, their daily routines have been enriched and the quality of family interactions has improved.

The research findings highlight the importance of developing training to design interventions that take into account their impact on child development and family quality of life. In Bruder and Dunst (2005) words, with a focus on accountability. The quality of training must be linked to the outcomes for the children and families for whom these services are intended.

Complementing the information collected with a qualitative perspective allows the voice of families to be heard, which should be a principle to work on in research that is interested in embracing the philosophy of family-centered practices.

## 6 Limitations and future directions

As with all research, our study has some limitations. The more important limitation with regard to the intervention with families is that, due to the time constraints of the students, there were no referrals to services that families and children need, such as appropriate medical care. There was also insufficient emphasis on families sending their children to inclusive centers, which is an aspect to consider both in professional training and in the characteristics of family-centered intervention.

The implementation of the RBI in the rural context of poverty proved to be efficient. However, it was not possible to go deeper into the characteristics of the families and the socio-economic context. In future research, there is a need for documentation and data collection that will allow a deeper understanding of the processes of adaptation of the RBI to the socio-economic and cultural diversity of Latin America.

## 7 Clinical implications

The results of the research also allow us to consider some clinical implications. The first has to do with the need to change the focus of interventions in highly vulnerable contexts, from proposing therapies aimed exclusively at the child, to promoting programs that empower families so that they are the ones to guide and support their children in the routines of daily life. The second is related to the need to bring these programs closer to the most geographically remote families, jointly between health and education services, so that they can continue throughout the children’s and families’ future schooling.

## Data availability statement

The raw data supporting the conclusions of this article will be made available by the authors, without undue reservation.

## Ethics statement

The studies involving humans were approved by Committee of Ethical Research – Universidad Casa Grande. The studies were conducted in accordance with the local legislation and institutional requirements. Written informed consent for participation in this study was provided by the participants’ legal guardians/next of kin. Written informed consent was obtained from the individual(s) for the publication of any potentially identifiable images or data included in this article.

## Author contributions

MF-J: Investigation, Methodology, Writing – original draft. MG: Conceptualization, Methodology, Supervision, Writing – review & editing.
